# What facilitates the delivery of dignified care to older people? A survey of health care professionals

**DOI:** 10.1186/s13104-015-1801-9

**Published:** 2015-12-28

**Authors:** Deborah Kinnear, Christina Victor, Veronika Williams

**Affiliations:** Institute of Health and Wellbeing, College of Medical, Veterinary and Life Sciences, University of Glasgow, Glasgow, Scotland, UK; College of Health and Life Sciences, Brunel University London, Uxbridge, UK; Health Experiences Research Group, Nuffield Department of Primary Care Health Sciences, University of Oxford, University of Oxford, Oxford, UK

**Keywords:** Dignity, Health care professionals, Older people, Dignified care, Facilitators, Barriers, Delivering care

## Abstract

**Background:**

Whilst the past decade has seen a growing emphasis placed upon ensuring dignity in the care of older people this policy objective is not being consistently achieved and there appears a gap between policy and practice. We need to understand how dignified care for older people is understood and delivered by the health and social care workforce and how organisational structures and policies can promote and facilitate, or hinder, the delivery of such care.

**Methods:**

To achieve our objective of understanding the facilitators and to the delivery of dignified care we undertook a survey with health and social care professionals across four NHS Trusts in England. Participants were asked provide free text answers identifying any facilitators/barriers to the provision of dignified care. Survey data was entered into SPSSv15 and analysed using descriptive statistics. These data provided the overall context describing staff attitudes and beliefs about dignity and the provision of dignified care. Qualitative data from the survey were transcribed verbatim and categorised into themes using thematic analysis.

**Results:**

192 respondents were included in the analysis. 79 % of respondents identified factors within their working environment that helped them provide dignified care and 68 % identified barriers to achieving this policy objective. Facilitators and barriers to delivering dignified care were categorised into three domains: ‘organisational level’; ‘ward level’ and ‘individual level’. Within the these levels, respondents reported factors that both supported and hindered dignity in care including ‘time’, ‘staffing levels’, training’,’ ‘ward environment’, ‘staff attitudes’, ‘support’, ‘involving family/carers’, and ‘reflection’.

**Conclusion:**

Facilitators and barriers to the delivery of dignity as perceived by health and social care professionals are multi-faceted and range from practical issues to interpersonal and training needs. Thus interventions to support health and social care professionals in delivering dignified care, need to take a range of issues into account to ensure that older people receive a high standard of care in NHS Trusts.

## Background

The NHS Constitution, enshrined in the 2009 Health Act, asserted that all patients have the right to be treated with dignity and respect, in accordance with their human rights. Whilst dignity is a contested and complex concept [1] a range of interventions and strategies to promote dignity in care have been developed in the UK. Organisational structures, culture, education, attitudes and behaviours of the health and social care workforce, at all levels ranging from managers to front line staff, are central to the successful implementation of strategies to promote dignity in care for older people in both hospital and community based care settings [2]. As Nolan [3] argues there are three key factors involved in the provision of dignified care to older people: the older person; family carers and professional staff. Research concerning dignity in care for older people has however predominately focused upon the experiences of patients, family and informal carers, with few studies focusing specifically on professional perspectives. Consequently there is a wealth of empirical evidence enumerating what patients and their relatives identify as the core elements of dignity in care namely: respect for personhood and the individual; communication and form of address; privacy; toileting; nutrition and feeding; cleanliness of environment; and staff attitudes [4–7]. These are the dimensions of dignity that regulatory bodies, policy makers and researchers have attempted to turn into measurable standards, practice guidelines, targets for achievement and inspection regimes [2, 8, 9]. However these concerns are not confined to the United Kingdom-researchers across a range of countries including Norway, Denmark and Sweden [10, 11] and China [12] have drawn attention to the challenges of delivering dignified care across a range of settings.

However the neglect of the professional perspective across studies focused upon dignity in care is striking and an important omission as it is staff that delivers care and is therefore crucial in the successful implementation of policies to deliver care within a dignified context. Indeed older people comment upon the centrality of staff behaviour and attitudes in the delivery of dignified care [13–15]. Policy documents argue for the need to change staff attitudes and behaviours as well as changing the culture of organisations delivering care [16]. It is the link (or gap) between policy and practice that is crucial for the patient’s experience of care: as Twigg [17] argues ‘It is at the front-line that the true nature of care reveals itself. It is there that it is created; and only there it can be judged’ (p. 1). Thus for dignified care to be delivered policies and practice need to be implemented at organisation, ward and individual level. However we have limited knowledge of the perspectives of health care professionals themselves or of the role of the organisational environment in shaping and contextualising the ability to deliver dignified care [18, 19]. The focus of our current evidence base is upon how staff understand and define dignified care and the importance they attribute to the concept [20]. This paper addresses a neglected but vital question of the perceptions of professional staff on the delivery of dignified care in practice and the identification of facilitators and barriers to delivery. This forms part of a larger case study (survey, interviews and focus groups) investigating how dignified care for older people is understood and delivered by health and social care professionals and; how organisational structures and policies can promote and facilitate, or hinder, the delivery of dignified care.

## Methods

### Dignity survey

To explore health and social care professionals’ perspectives and experiences of dignified care we developed a self-completion questionnaire. This consisted of 22 questions, exploring health and social care professionals’ perspectives and experiences of dignified care and was modelled on the instrument developed by the Royal College of Nursing report [21] and informed by research which had taken place since the completion of that survey [22]. Overall, 50 % of our instrument was based on the RCN survey providing a comparative context for our study. Both closed and open-ended questions were used to gain an insight into the experiences and perspectives of health and social care professionals. In addition, participants were asked to provide standard demographic data (gender, age, ethnicity and job role). The purpose of these details was to evidence the characteristics of the participant population and to be able to consider how representative our sample was of the general health care providing population. Participants were also given the opportunity at the end of the questionnaire to provide any further comments on providing dignified care for older people.

### Pilot study

We piloted the questionnaire with health professionals from one of the project sites where ethical approval had been received. The research fellow (DK) visited the site and provided the lead nurse of patient experience research, who was assisting with recruitment, with questionnaires (n = 10) for health and social care staff to pilot. In addition, health and social care professionals (n = 5) known to the research fellow were invited to provide feedback on the questionnaire. Minor changes to font and layout were made following this exercise. No new items were added providing evidence for the face validity of the tool. However developing an on-line version of the measure was suggested to widen the potential scope for participation and this was adopted.

### Participants and procedure

Data were collected for 12 months between June 2011 and June 2012. Ethical approval for the study was obtained (REC ref number: 10/H0711/49) from both Brunel University and the UK National Research Ethics Service (NRES).

The participants in this study were drawn from health and social care professionals aged 18 years or over working within four NHS Trusts (two acute trusts, one mental health trust and one primary care trust) in England and who provided care for older people. The premise was that it was of more value to conduct our research in settings where there was a ‘good’ culture of dignified care so that we could learn from these rather than look at settings that were doing less well. This allowed us to explore issues relating to the delivery of dignified care in various organizational and care settings as well as across profession (i.e. nursing, medical staff, social work, allied health professions) groups and grade specific groups of staff.

Gatekeepers were identified within each trust who assisted with recruitment of health and social care professionals. Packs containing a letter of invitation to take part in the study, an information leaflet, a survey, a consent form to take part in the in-depth interviews, a small stamped addressed envelope for the consent form and a large stamped addressed envelope to return the survey were provided to professionals who met the inclusion criteria. Separate envelopes were provided to ensure that participants who completed the survey were not identified from the information they provided on the consent form. The main researcher (DK) visited staff in the primary care trust and all wards in both acute trusts where older people were cared for in some capacity and spoke with staff before leaving packs of surveys and informing them about the online survey. Additional surveys were left with ward managers and placed in staff rooms. For the mental health trust, packs of surveys were delivered by post to the main contact who then administered these to appropriate staff members.

### Analysis

#### Dignity survey

Data were cleaned and analysed using the Statistical Package for the Social Sciences (SPSS) version 18 and analysed using descriptive statistics in order to elucidate professionals’ understandings of the facilitators and barriers to delivering dignified care. For the qualitative data within the survey a content analysis was used which is appropriate for a descriptive approach focussed upon attaining an overview of how our participants understood the phenomena of dignity [23].

## Results

### Response rate

Overall 650 hard copies of surveys were administered across three of the four NHS trusts, of which 161 were returned (25 % response rate). All four trusts received an email invitation with a link to take part in the online survey and 31 questionnaires were completed online giving a total of 192 completed surveys. Our original plan had been to explore variations in staff attitudes and beliefs about dignity and the provision of dignified care across settings, professional groups and demographic factors such as age and gender. However the response rate and resultant sample size (n = 192) precluded this comparative analysis and our quantitative analysis is therefore presented for the sample as a whole. We integrate the results of both elements of our study to provide an integrated analysis of the facilitators and barriers to providing dignified care.

### Profile of participants

The majority of participants were female (86 %) and 82 % were aged between 25 and 54 years. The ethnicity of participants was diverse but the majority (n = 134) described themselves as White British. Participants were asked to select their job role from a list of options of which 61 (32 %) participants selected the category ‘staff nurse’. Participants who selected the category ‘other’ (n = 58) were asked to provide additional details about their job role: 45 reported that they had a nursing background such as sister, matron, ward sister, ward manager, junior sister, research nurse and specialist cancer nurse. The remaining 13 participants included four psychologists and one each of a diverse range of roles (e.g. occupational therapy assistant; radiographer, podiatrist, psychiatrist, physiotherapy assistant and social work assistant). The remaining roles cannot be reported as they were unique to the trust and thereby rendering participants potentially identifiable. Overall, 109 (57 %) of the participants had a nursing background.

### Delivering dignified care

Before considering the factors underpinning the delivery of dignified care we asked participants to rate their own practice. A quarter of respondents felt that they delivered dignified care all of the time and 67 % reported that they were able to deliver dignified care most of the time.

We asked participants how easy (or difficult) it was for them to deliver specific aspects of dignified care. Having time to promote patients autonomy, providing adequate information, listening to patients whilst providing care and maintaining privacy whilst providing this were rated as ‘easy’ to deliver by two thirds (or more) of our respondents (see Table 1). Two problem areas stand in terms of being barriers to delivering dignified care: availability of quiet rooms and providing help with meals. The availability of quiet rooms to talk to patients and relatives reflects an infrastructure barrier resultant from the design of wards. Help at meals times reflected the fact that some participants commented that they did not help with meals because it was not ‘their job’ (p. 176) or this role was “not applicable to nursing staff. Health care assistants help” (p. 158). Others highlighted staffing difficulties as these comments illustrate: “…some patients receive food cold as not enough staff to assist patients (all) at once” (p. 8); “I can only feed one person at a time!’’ (p. 39) and “sometimes patients have cold food cold-not enough staff” (p. 40). Here the barriers to delivering dignified care reflect professional responsibilities and the clarifications of roles and the availability of staffing/time.

**Table 1:** Ease of delivering dignified care, in %

Task	Easy	Neither easy nor difficult	Difficult
Maintaining privacy when providing personal care	60	27	13
Providing help with meals	30	57	13
Access to side rooms to talk to patients in privacy	30	32	38
Provision of a clean care environment	60	31	9
Having time to talk and actively listen to patients when delivering care	60	22	18
Providing adequate information to patients about their care	76	18	6
Ability to promote patients’ autonomy and right to make independent choices	74	24	4

### Facilitators and barriers to delivering dignified care

#### Facilitators

Overall 79 % (n = 152) participants reported that there were factors in their work setting that supported them in providing dignified care. 11 % (n = 22) participants stated that there were no factors that helped them deliver dignified care and 9 % (n = 18) responded that they did not know. Those participants reporting the presence of factors that helped them provide dignified care they were asked to provide a free text response describing what these were. A number of themes emerged of the factors that helped health and social care staff provide dignified care which were categorised into three domains: ‘Organisational level’; ‘Ward level’ and ‘Individual level’ as our project sought to identify the relative importance of these 3 domains in promoting the delivery of dignified care (see Table 2). However for some respondents they provided information about what factors would help them deliver dignified care rather than what factors actually do help them deliver dignified care. This was particularly evident with responses in relation to ‘time’, ‘staffing levels’, ‘resources’ and ‘work load’ as these examples illustrate:

**Table 2:** Facilitators and barriers to the delivery of dignified care at the organisational, ward and individual level

	Facilitators	n	Barriers	n
Organisational level				
Time	“Having time to allow patients to express their thoughts/fears” (p. 28)	42	“Not enough time!” (p . 23)	47
Staffing levels	“Better staffing on the wards!” (p. 131)	34	“Inadequate staffing levels” (p. 13)	37
Staff training and experience	“Putting training into practice and delivering dignified care” (p. 11)	26	“Poorly trained/incompetent nurses” (p. 133)	11
Organisational support/values	“Having the right culture within the organisation with regard to values” (p. 42)	7	“The quantity of patient turnover and meeting target times that often appears more important to certain staff (i.e. managers)” (p. 33)	13
Resources	“Having the right resources to provide the right support and care (staff and equipment)” (p. 42)	19	“Lack of equipment” (p. 68)	19
Specific Dignity measures	“Protected meal times” (p. 89)	6	“Staff entering curtained area despite ‘privacy peg’” (p. 32)	11
Ward level				
Ward environment	“Mobilising equipment to toilet/bathroom wherever possible rather than using commodes/bed pans” (p. 30)	23	“Lack of appropriate facilities such as quiet rooms to discuss confidential issues” (p. 29)	26
Colleagues/team	“Excellent teams within care of the elderly. Consultants are very proactive as are senior nurses and ward staff” (p. 61)	21	“Poor staff mix” (p. 182)	2
Staff attitudes	“Other members of staff participation in dignified care, everyone having the same ‘goal’” (p. 16)	13	“Non empathetic nurses” (p. 74)	8
Work load	“Less workload” (p. 58)	7	“Pressure to do things in a hurried manner can lead to a loss of dignity” (p. 179)	30
Support	“Support from colleagues” (p. 133)	19	“Not being supported by staff” (p. 45)	7
Communication	“Good communication between myself and members of the multi-disciplinary team” (p. 19)	4	“Staff not communicating well” (p. 130)	8
Individual level				
Addressing patients needs	“An understanding of specific patient needs or beliefs” (p. 94)	17	“Sometimes feel we are box-filling and patients/carers may not feel this is individualised” (p. 73)	8
Involving family/carers	“Involving family/carers—this also provides an opportunity to gain insight into patient circumstances” (p. 5)	3	n/a	
Reflection	“Having the time to reflect on my own practice and prejudices and challenge my thinking about what ageing is about” (p. 101)	1	n/a	
Dealing with an emergency	n/a		“Emergency situations when the focus is on saving lives (if in patients best interest)—sometimes it is difficult to prevent exposure/timely communication” (p. 7)	3
Religion	n/a		“Religion can prevent you from providing dignified care although it shouldn’t. For example a muslim woman cannot be cared for by a male nurse because of their faith” (p. 53)	1
Neglect	n/a		“Staff not answering patients buzzers resulting in wetting the bed due to long wait” (p. 173)	1

“Having enough time and staff to give you half the chance to listen to what a patient needs rather than guessing because you are so swamped with work!” (p. 180)

“IF [bold and underlined by participant] we have enough staff on shift then care is more dignified because patients are not waiting as long for meals, hygiene needs and we have more time to listen to our patients’ needs” (p. 11)

“To have the correct facilities and tools to be able to carry out care such as specific questions on assessment forms to indicate gender of carer to help them with washing and dressing for example” (p. 116)

Facilitators at ward level focussed upon the staff team working in the specific care environment, especially around shared values and support for each other. Few comments were made about the role of organisation specific dignity delivery measures in supporting this policy goal.

#### Barriers

A total of 130 (68 %) participants stated that there were factors that prevented them from providing dignified care, 38 (20 %) stated there were no factors and 24 (12 %) stated that they did not know. If participants stated that there were factors that prevented them from providing dignified care they were asked to specify what these were. Emerging themes were once again classified into the following three domains: ‘Organisational level’; ‘Ward level’ and ‘Individual level’ (see Table 2). Some, but not all, factors identified were the opposite of the factors that were also facilitators such as lack of time, staffing levels, workload and limited resources. However job pressures and the physical nature of the ward environment were also noted as factors that could militate against the provision of dignified care.

Comparison of the relative importance of barriers and facilitators across the same themes illuminates the complexity of trying to understand how the provision of dignified care can be best supported in practice. At the organisational level ‘time’, staffing levels and organisational support are predominantly barriers rather than facilitators whilst for training this is seen as being a key positive organisational level of support for providing dignified care. At ward level workload clear impacts upon perceived ability to dignified care and the nature of the ward environment is equally important in both categories. Key for our participants in supporting the provision of dignified care at ward level were the working relationships they had with colleagues/team their attitudes and the support they received from colleagues. Part from addressing patient needs few individually focussed factors were identified in supporting the provision of dignified care.

### Organisational level support for providing dignified care

We explicitly asked participants in the survey to rate how their organisation supported the delivery of care in a range of ways (Table 3). Training in dignified care either at induction or for existing staff was reported by staff as being provided and the provision of such care was seen as being integral to Trust policies promoting dignity. Staff also felt that the organisation supported the delivery of dignified care by the provision of a confidential reporting system for when breeches in this were observed. Resources, as noted earlier were seen as important to dignified care provision and our participants were broadly supportive of the skill mix but not staffing levels.

**Table 3:** Participants’ rating of their organisation in supporting the delivery of dignified care

Employer support	Yes	Somewhat	No	Don’t know
Dignified care in new staff induction	98	18	13	63
Internal training on dignity	96	25	28	43
Work philosophy mentions dignity	120	22	15	35
Good skill mix	109	73	8	2
Good staffing levels	44	108	40	0
Discuss difficult issues of dignity with colleagues	121	58	11	2
Include dignity in care when teaching/working with students/new staff	153	35	2	2
Feel able to report breaches of dignity in care in confidence to my manager/employer	123	52	11	6

Participants were provided with a list of organisational level options to maintain and promote dignified care (see Table 4) and invited to identify the three most important options to help them maintain and improve their ability to provide dignified care. In line with evidence from previous chapters-resources-in terms of the inter-linked issues of staffing, time and less work pressures, were seen as the key to helping them maintaining and improving dignified care. These are generic solutions which do not relate specifically to the idea of dignity but which would have consequences across a range of aspects of care delivery. Support from managers, peers and a better working environment were rated as much less important: time, staff and less work pressures were seen as key to the delivery of dignified care.

**Table 4:** Participants’ ratings of the most important options to help them maintain and improve their ability to provide dignified care

Which of these would help you to maintain and improve your ability to provide dignified care? (rank top 3 most important, 1 = most important, 8 = least important)	Most important
Better staffing	1
More time	2
Less work pressure	3
Education	4
Integration of dignity into work philosophy	5
Support from managers/organisation	6
Better work environment	7
Peer support	8

## Discussion

Our study sought to develop the debate about delivering dignified care in two key ways by focussing upon the facilitators and conceptually by looking for factors operating at organisational, ward and individual level. These domains resonate closely with the three areas that have the potential to improve or diminish dignity in care according to The Royal College of Nursing (RCN) [21]. Defending dignity—challenges and opportunities for nursing: people—the attitudes and behaviour of staff and visitors; place—the physical environment and the culture of the employing organisation and; processes—sensitivity and attention to the wide range of care activities which could threaten dignity [8]. Further, part of the RCN’s [21] definition of dignity states that:

“In care situations, dignity may be promoted or diminished by: the physical environment; organisational culture; by the attitudes and behaviour of nurses and others and by the way in which care activities are carried out”.

This paper has considered the importance of dignified care delivery to a range of health care practitioners and has sought to identify the facilitators and barriers they perceive to delivering dignified care at three levels of care delivery: the individual, the ward environment and the organisational level. Our study had sought to determine the positive (and negative) factors that supported (or hindered) the delivery of dignified care and the organisational mechanisms to support it. We have already noted the greater emphasis in our participants’ narratives on the barriers as opposed to the supportive factors (see Table 2). We may speculate that it is easier to identify and talk about barriers-as they are obvious and identifiable-whereas facilitators may be less explicit and embedded within the taken for granted routines and activities of daily practice. This distinction has not been raised in previous studies and, perhaps, we need to adopt more innovative research techniques such as vignettes or case scenarios to try to tease out the factors that support dignified care.

For the factors that facilitated dignity that emerged from our study there are two key points to note. First, as Table 5 illustrates, factors that facilitated dignified care were predominately situated at the ward and individual level encompassing strong and positive staff attitudes; strong ward leadership, cohesive teams with good communication; the general working protocols and the adoption of dignity specific initiatives. Tadd et al. [22] also note the importance of the ward-or care location-in promoting (or hindering) the delivery of dignified care. Barriers to care delivery were seen as operating at the macro level in terms of both the organisation and societal level. Our participants also felt that contemporary nursing education did not support the delivery of dignified care and reflects wider debates about the education of health and social care professionals in terms of the balance between theory and practice based education. Ours is the first study to suggest the importance of societal views about the value of caring and older people in acting as barriers to the delivery of dignified care. This finding merits further investigation.

**Table 5:** Facilitators and barriers to dignified care delivery by level of analysis

	Organisation	Ward	Individual	Societal
Facilitating dignity				
Protocols and procedures		×		
Staff attitudes and values		×	×	
Staff mix, communication and culture and team working		×		
Adoption of interventions to promote dignity		×		
Barriers to dignified care				
Resources-staffing, time, skill mix, task orientated care	×			
Culture and values-including valuing staff	×			
Workforce diversity	×			
Inadequate/routine education about dignity	×			
Poor communication			×	
Working on auto-pilot			×	
Value of care and caring				×
Valuing and prioritisation of older people				×
Failures of nursing education				×

Summarising our findings we see that the factors supporting the delivery of dignified care were predominantly located at the ward level and individual level and focussed upon the staff team and their skills and attributes (see Table 5). Similar findings were reported in a literature review by Gallagher [24] who critically reviewed the theoretical and empirical literature relating to dignity and clarifying the meaning and implications of dignity in relation to the care of older people. Gallagher et al. identified four key and recurrent themes, two of which included: ‘The environment of care’ and ‘Staff attitudes and behaviour’. ‘The environment of care’ related to physical features of the environment which had the potential to either facilitate or hinder dignified care such as privacy in care and access to bathroom and toilet facilities. ‘Staff attitudes and behaviour’ related to the way individuals responded to patients and others in the language they used such as ‘darling’ and ‘poppet’ and in their actions such as dressing and grooming patients in a way they wished. It was clear from the analysis in the current study that staff relationships with one another appeared to be central to facilitating dignified care. A number of staff, for example, reported the importance of working as part of a team, having support from other colleagues and working alongside staff who had positive attitudes about delivering dignified care. These findings are reassuring as the importance of teamwork within a health care setting has been stressed in previous research, for example, in achieving patient safety [25], job satisfaction [26, 27] and quality of care [28]. However, Kalisch and Lee [29] suggest that when nursing staff, for example, are stressed and overwhelmed by their work load due to insufficient staff, teamwork decreases. Developing and maintain teamwork is clearly an important dimension of providing dignified care.

At the Organisational level high patient turnover, meeting targets, lack of time, resources and insufficient staffing levels were articulated as barriers to delivering dignified care. This is of concern, as the inability to deliver appropriate standards of care because of systemic or organisational factors results in a struggle to provide not only continuity of care, but care that protects and promotes the individual’s dignity [30]. Staff shortages, for example, has previously been reported as adversely affecting dignity in care [31, 32] as have insufficient resources [13, 16, 17]. However, as our participants’ indicated, the important element is having not just a specific number of staff but also the ‘right’ sort of staff. Thus debates about staffing levels and their relationship with the delivery of dignified care are inevitably superficial if no account is taken of the quality/skills of staff. It is therefore vital that staff wishing to provide dignified care for patients feel supported when they identify areas which may have a detrimental impact on their patients’ care, such as poor staffing levels and lack of appropriate equipment, as described in the current study.

At the Individual level participants in the current study reported identifying and addressing patients specific and individual needs as a facilitator to delivering dignified care, an area which the Royal College of Nursing [21] emphasises as a core nursing value: “To treat someone with dignity is to treat them as being of worth, in a way that is respectful of them as valued individuals”. However, some staff (n = 8) felt that they were unable to provide individualised care to patients due to an increase in box-ticking, performance targets, limited staff and time with patients. The Picker Institute’s survey of older people’s dignity reiterates the possible threat from increasing performance targets together with reduced staff ratios and found process targets and budgetary concerns militate against professionals delivering dignified care [33]. While the remaining themes within the Individual level were mentioned by very few, they were considered important to include. Having the time to reflect was reported as a facilitator to delivering dignified care and has previously been recommended as an important area for health care training, continuing education and medical audit programmes [34–36].

It is worth noting factors supporting/compromising the delivery of dignified care that our participants did not articulate. Unlike previous studies [24] the characteristics of patients, especially in terms of frailty, dementia and multiple pathology, were not cited as explicit factors that compromised staff ability to deliver dignified care. Previous research has identified the ‘right patient-wrong place’ narrative in explanations for problems in delivering dignified care specifically and good quality care more generally. This omission in our study may reflect the specific characteristics of the patient population in our research settings or the ‘self-selected’ nature of our research participants who may have been more experienced in dealing with these issues. Alternatively participants may have focussed upon ‘external’ organisational and broader societal factors when considering the things that precluded the delivery of dignified care. It is also important to note that the facilitators of care are not simply the obverse of barriers because of the different operational level they were identified at. Again further research is warranted into how these factors engage across the different levels of health and social care delivery systems to promote the delivery of dignified care.

## Limitations

Our findings come with some limitations. Whilst we have a large absolute sample this represents at best about a third of the total study population. However, similar findings were reported by the RCN [21] who attracted only a small fraction (n = 2048) of their total membership (n = 600,000). It is also unclear as to whether these findings represent those most engaged with the dignity agenda. In terms of gender and ethnicity, our sample reflects the general NHS health care professional population [21].

## Conclusion

Our study highlights the facilitators and barriers to delivering dignified care. We were partially successful in our aim of identifying factors that promote the delivery of dignified care. Our participants found it much more difficult to comment on the positive than the negative. We specifically wanted to identify what supports dignified care delivery. As such we opted to conduct our study in care settings with excellent ‘dignity ratings’ to try to articulate supportive factors. However our participants found this difficult to articulate in an abstract research context. This suggests that we need to develop other ways of investigating this such as vignette based studies or case reviews or critical incident analysis of cases or examples of dignified care.

And dignified in order to address the barriers that health and social care professionals’ face, proactive measures are required to ensure that dignified care is delivered. One such example is a recent Department of Health report Compassion in Practice: Nursing, Midwifery and Care Staff—Our vision and Strategy’ [37] which sets out to deliver high quality, compassionate care, and to achieve excellent health and wellbeing outcomes. Over 9000 nurses, midwives, care staff and patients took part in an engagement exercise with clear messages shaping the vision and strategy in this document. The vision was underpinned by six fundamental values: care, compassion, competence, communication, courage and commitment—with six areas of action (e.g. building and strengthening leadership) to support professionals and care staff to deliver dignified and excellent care. One step to implement this vision and strategy is to work closely with regional and front line staff to understand the barriers that need to be addressed and overcome, so that this vision does reach the heart of every care setting and makes a positive and sustained difference to the people they care for.

While there are many issues raised in this paper on the delivery of dignified care for older people it must be stressed that there are also positive and encouraging signs that dignified care is being delivered within these NHS Trusts. However, it is apparent that there are clear failures within the system that threaten the delivery of dignified care such as inadequate staffing, lack of time to spend with patients, heavy workload and lack of resources to name a few. As emphasised by Oliver [36], constructive, effective solutions require multiple approaches and go beyond ‘knee-jerk’ blaming of clinical staff. Furthermore we would argue that understanding the delivery of dignified care requires a broader focus than individual staff or ward settings. We need to develop analyses that focus upon organisational levels of activity and the wider social context. Until value working with older people positively then the delivery of dignified care will always present a challenge for both individuals and organisations.
